# Risk for Transportation of 2019 Novel Coronavirus (COVID-19) from Wuhan to Cities in China

**DOI:** 10.1101/2020.01.28.20019299

**Published:** 2020-02-17

**Authors:** Zhanwei Du, Lin Wang, Simon Cauchemez, Xiaoke Xu, Xianwen Wang, Benjamin J. Cowling, Lauren Ancel Meyers

**Affiliations:** 1. The University of Texas at Austin, Austin, Texas 78712, The United States of America; 2. nstitut Pasteur, 28 rue du Dr Roux, Paris 75015, France; 3. Dalian Minzu University, Dalian 116600, China.; 4. Dalian University of Technology, Dalian 116024, China; 5. The University of Hong Kong, Sassoon Rd 7, Hong Kong SAR, China; 6. Santa Fe Institute, Santa Fe, New Mexico, The United States of America

**Keywords:** Wuhan, coronavirus, epidemiology, importation

## Abstract

On January 23, 2020, China quarantined Wuhan to contain an emerging coronavirus (COVID-19). We estimated the probability of transportation of COVID-19 from Wuhan to 369 cities in China before the quarantine. The expected risk is >50% in 130 (95% CI 89–190) cities and >99% in the 4 largest metropolitan areas of China.

In December 2019, a novel coronavirus (COVID-19) emerged in Wuhan, China (1). On January 30, 2020, the World Health Organization (WHO) declared the outbreak a public health emergency of international concern (2). By January 31, 2020, 192 fatalities and 3,215 laboratory-confirmed cases were reported in Wuhan; 8,576 additional cases were spread across >300 cities in mainland China; and 127 exported cases were reported in 23 countries/states spanning Asia, Europe, Oceania, and North America. The rapid global expansion, rising fatalities, unknown animal reservoir, and evidence of person-to-person transmission potential (3,8) initially resembled the 2003 SARS epidemic and raised concerns about global spread.

On January 22, 2020, China announced a travel quarantine of Wuhan and by January 30, expanded the radius to include 16 cities, encompassing a population of 45 million. At the time of the quarantine, China was already 2 weeks into the 40-day Spring Festival, during which several billion people travel throughout China to celebrate the Lunar New Year (4). Considering the timing of exported COVID-19 cases reported outside of China, we estimate that only 8.95% (95% CrI 2.22% - 28.72%) of cases infected in Wuhan by January 12 might have been confirmed by January 22, 2020. By limiting our estimate to infections occurring ≥10 days before the quarantine, we account for an estimated 5–6-day incubation period and 4–5 days between symptom onset and case detection (3,5,6,8) (Appendix). The low detection rate coupled with an average lag of 10 days between infection and detection (6) suggest that newly infected persons who traveled out of Wuhan just before the quarantine might have remained infectious and undetected in dozens of cities in China for days to weeks. Moreover, these silent importations already might have seeded sustained outbreaks that were not immediately apparent.

We estimated the probability of transportation of infectious COVID-19 cases from Wuhan to cities throughout China before January 23 by using a simple model of exponential growth coupled with a stochastic model of human mobility among 369 cities in China (Appendix). Given that an estimated 98% of all trips between Wuhan and other Chinese cities during this period were taken by train or car, our analysis of air, rail, and road travel data yields more granular risk estimates than possible with air passenger data alone (7).

By fitting our epidemiologic model to data on the first 19 cases reported outside of China, we estimate an epidemic doubling time of 7.31 days (95% CrI 6.26 – 9.66 days) and a cumulative total of 12,400 (95% CrI 3,112–58,465) infections in Wuhan by January 22, 2020 (Appendix). Both estimates are consistent with a recent epidemiologic analysis of the first 425 cases confirmed in Wuhan (8). By assuming these rates of early epidemic growth, we estimate that 130 cities in China have ≥50% chance of having a COVID-19 case imported from Wuhan in the 3 weeks preceding the quarantine (Figure). By January 26th, 107 of these high-risk cities had reported cases and 23 had not, including 5 cities with importation probabilities >99% and populations >2 million: Bazhong, Fushun, Laibin, Ziyang, and Chuxiong. Under our lower bound estimate of 6.26 days for the doubling time, 190/369 cities lie above the 50% threshold for importation. Our risk assessment identified several cities throughout China likely to be harboring yet undetected cases of COVID-19 a week after the quarantine, suggesting that early 2020 ground and rail travel seeded cases far beyond the Wuhan region under quarantine.

Our conclusions are based on several key assumptions. To design our mobility model, we used data from Tencent (https://heat.qq.com), a major social media company that hosts applications, including WeChat (≈1.13 billion active users in 2019) and QQ (≈808 million active users in 2019) (9); consequently, our model might be demographically biased by the Tencent user base. Further, considerable uncertainty regarding the lag between infection and case detection remains. Our assumption of a 10-day lag is based on early estimates for the incubation period of COVID-19 (8) and prior estimates of the lag between symptom onset and detection for SARS (10). We expect that estimates for the doubling time and incidence of COVID-19 will improve as reconstructed linelists and more granular epidemiologic data become available (Appendix). However, our key qualitative insights likely are robust to these uncertainties, including extensive pre-quarantine COVID-19 exportations throughout China and far greater case counts in Wuhan than reported before the quarantine.

## Supplementary Material

1

## Figures and Tables

**Figure 1.**
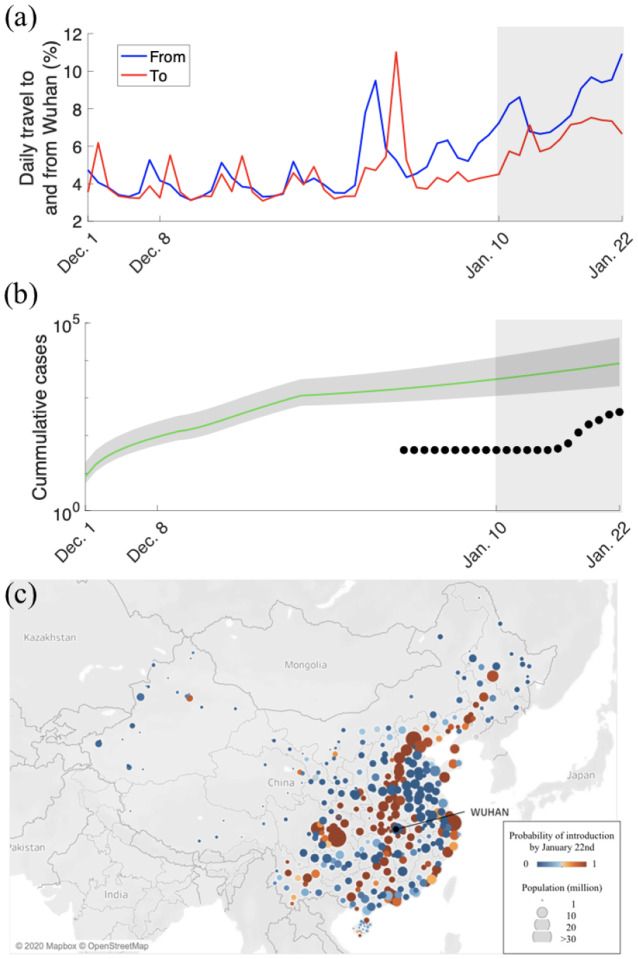
Risks for COVID-19 transportation from Wuhan, China before a quarantine was imposed on January 23, 2020. a) Daily travel volume to and from Wuhan, given as a percentage of the Wuhan population. The shading on the right indicates the start of Spring Festival season on January 10, 2020, which is a peak travel period in China. b) Estimated and reported daily prevalence of COVID-19 in Wuhan. The green line and shading indicate model estimates of cumulative cases since December 1, 2019 with 95% CrI bounds, based on an epidemic doubling time of 7.38 days (95% CrI 5.58–8.92 days). Black points are cumulative confirmed case counts during January 1–22, 2020 (11). The shading on the right indicates the start of Spring Festival season. c) Map generated by using Mapbox (https://www.mapbox.com) representing the probability that ≥1 COVID-19 case infected in Wuhan traveled to cities in China by January 22, 2020. The 131 cities above a risk threshold of 50% are indicated in orange; the 239 cities below the threshold are indicated in blue.
