# Supplementary material for: Risk for Transportation of 2019 Novel Coronavirus (COVID-19) from Wuhan to Cities in China
Source: medRxiv. 2020 Feb 17:2020.01.28.20019299. Preprint. [Version 4] doi: 10.1101/2020.01.28.20019299 (PMC7239085; doi:10.1101/2020.01.28.20019299)
Supplement: 1 [file NIHPP2020.01.28.20019299-supplement-1.pdf]

medRxiv preprint doi: https://doi.org/10.1101/2020.01.28.20019299.this version posted February 17, 2020. The copyright holder for this preprint
(which was not certified by peer review) is the author/funder, who has granted medRxiv a license to display the preprint in perpetuity.
It is made available under a CC-BY-NC-ND 4.0 International license .

Supplementary Appendix

Data

We analyzed the daily number of passengers traveling between Wuhan and 369 other cities in mainland China. We obtained mobility data from the location-based services of Tencent (https://heat.qq.com). Users permit Tencent to collect their real-time location information when they install applications, such as WeChat (≈1.13 billion active users in 2019) and QQ (≈808 million active users in 2019), and Tencent Map. By using the geolocation of users over time, Tencent reconstructed anonymized origin–destination mobility matrices by mode of transportation (air, road, and train) between 370 cities in China, including 368 cities in mainland China and the Special Administrative Regions of Hong Kong and Macau. The data are anonymized and include 28 million trips to and 32 million trips from Wuhan, during December 3, 2016–January 24, 2017. We estimated daily travel volume during the 7 weeks preceding the Wuhan quarantine, December 1, 2019–January 22, 2020, by aligning the dates of the Lunar New Year, resulting in a 3-day shift. To infer the number of new infections in Wuhan per day during December 1, 2019–January 22, 2020, we used the mean daily number of passengers traveling to the top 27 foreign destinations from Wuhan during 2018–2019, which were provided in other recent studies (*1*–*3*).

Model

We considered a simple hierarchical model to describe the dynamics of 2019 novel coronavirus (COVID-19) infections, detections, and spread.

medRxiv preprint doi: https://doi.org/10.1101/2020.01.28.20019299.this version posted February 17, 2020. The copyright holder for this preprint
(which was not certified by peer review) is the author/funder, who has granted medRxiv a license to display the preprint in perpetuity.
It is made available under a CC-BY-NC-ND 4.0 International license .

Epidemiologic Model

By using epidemiologic evidence from the first 425 cases of COVID-19 confirmed in Wuhan by January 22, 2020 (*4*), we made the following assumptions regarding the number of new cases, $dI_w(t)$, infected in Wuhan per day, t.

- The COVID-19 epidemic was growing exponentially during December 1, 2019–January 22, 2020, as determined by the following:

  $$dI_w(t) = i_0 \times \exp(\lambda \times t)$$

  in which i_0 denotes the number of initial cases on December 1, 2019 (*5*), and λ denotes the epidemic growth rate during December 1, 2019–January 22, 2020.

- After infection, new cases were detected with a delay of D = 10 days (*6*), which comprises an incubation period of 5–6 days (*4,7–11*) and a delay from symptom onset to detection of 4–5 days (*12,13*). During this 10-day interval, we labeled cases as infected. Given the uncertainty in these estimates, we also performed the estimates by assuming a shorter delay (D = 6 days) and a longer delay (D = 14 days) between infection and case detection (Appendix Table 2).

Our model can be improved by incorporating the probability distribution for the delay between infection and detection, as reconstructed linelists (*14–17*) and more granular epidemiologic data are becoming available.

Under these assumptions, we calculated the number of infectious cases at time, t, by the following:

$$I_\omega(t) = \int_{u=t-D}^{t} dI_\omega(u)du$$

The prevalence of infectious cases is given by the following:

$$\xi(t) = \frac{I_\omega(t)}{N_\omega}$$

in which $N\omega$ = 11.08 million, the population of Wuhan.

medRxiv preprint doi: https://doi.org/10.1101/2020.01.28.20019299.this version posted February 17, 2020. The copyright holder for this preprint (which was not certified by peer review) is the author/funder, who has granted medRxiv a license to display the preprint in perpetuity.
It is made available under a CC-BY-NC-ND 4.0 International license .

Mobility Model

We assume that visitors to Wuhan have the same daily risk for infection as residents of Wuhan and construct a nonhomogeneous Poisson process model (*18–20*) to estimate the exportation of COVID-19 by residents of and travelers to Wuhan. Let $W_{j,t}$ denote the number of Wuhan residents that travel to city *j* at time *t*, and $M_{j,t}$ denote the number of travelers from city *j* to Wuhan at time *t*. Then, the rate at which infected residents of Wuhan travel to city *j* at time *t* is given $\gamma_{j,t} = \xi(t) \times W_{j,t}$, and the rate at which travelers from city *j* get infected in Wuhan and return to their home city while still infected is $\Psi_{j,t} = \xi(t) \times M_{j,t}$. This model assumes that newly infected visitors to Wuhan will return to their home city while still infectious.

Based on this model, the probability of introducing ≥1 cases from Wuhan to city *j* by time *t* is given by

$$1 - exp\left[- \int_{u=t_0}^{t} (\gamma_{j,u} + \Psi_{j,u}) du \right]$$

where t_0 denotes the beginning of the study period, December 1, 2019.

Inference of Epidemic Parameters

We applied a likelihood-based method to estimate our model parameters, including the number of initial cases i_0 and the epidemic growth rate λ, from the arrival times of the 19 reported cases transported from Wuhan to 11 cities outside of China, as of January 22, 2020 (Appendix Table 1). All 19 cases were Wuhan residents. We aggregated all other cities without cases reported by January 22, 2020 into a single location ($j = 0$).

Let N_j denote the number of infected Wuhan residents who were detected in location *j* outside of China, $x_{j,i}$ denote the time at which the *i*-th Wuhan resident case was detected in location *j*, $\chi_{j,0}$ denote the time at which international surveillance for infected travelers from Wuhan began (January 1, 2020) (*21*), and *E* denote the end of the study period (January 22, 2020). As indicated above, the rate at which infected residents of Wuhan arrive at location *j* at

medRxiv preprint doi: https://doi.org/10.1101/2020.01.28.20019299.this version posted February 17, 2020. The copyright holder for this preprint
(which was not certified by peer review) is the author/funder, who has granted medRxiv a license to display the preprint in perpetuity.
It is made available under a CC-BY-NC-ND 4.0 International license .

time t is $\gamma_{j,t}$. Then, the likelihood for all 19 cases reported outside of China by January 22, 2020 is given by

$$\prod_{j=0}^{11} \exp(-\int_{x_{j,N_j}}^{E} \gamma_{j,t} dt) \prod_{i=1}^{N_j} \gamma_{j,x_{j,i}} \exp(-\int_{x_{j,i-1}}^{x_{j,i}} \gamma_{j,t} dt) .$$

which yields the following log-likelihood function:

$$\sum_{j=1}^{11} \sum_{i=1}^{N_j} \log(\gamma_{j,x_{j,i}}) - \frac{\sum_{j=0}^{11} W_{j,t}}{N_w} \times \frac{i_0}{\lambda^2} \times [\exp(\lambda \times E) - \exp(\lambda \times x_{j,0})$$

$$+ \exp(\lambda \cdot (x_{j,0} - D)) - \exp(\lambda \cdot (E - D))]$$

Parameter Estimation

We directly estimated the number of initial cases, i_0, on December 1, 2019, and the epidemic growth rate, λ, during December 1, 2019–January 22, 2020. We infer the epidemic parameters in a Bayesian framework by using the Markov Chain Monte Carlo (MCMC) method with Hamiltonian Monte Carlo sampling and noninformative flat prior. From these, we derive the doubling time of incident cases as $d_T = \log(2)/\lambda$ and the cumulative number of cases and of reported cases by January 22, 2020. We also derived the basic reproduction number by assuming a susceptible-exposed-infectious-recovery (SEIR) model for COVID-19, in which the incubation period is exponentially distributed with mean L in the range of 3 - 6 days and the infectious period is also exponentially distributed with mean Z in the range of 2 to 7 days. The reproduction number is then given by $R_0 = (1 + \lambda \times L) \times (1 + \lambda \times Z)$.

We estimated the case detection rate in Wuhan by taking the ratio between the number of reported cases in Wuhan by January 22, 2020 and our estimates for the number of infections occurring ≥10 days prior (i.e., by January 12, 2020). We truncated our estimate 10 days before the quarantine to account for the estimated time between infection and case detection, assuming a 5–6 day incubation period (*4,7–11*) followed by 4–5 days between symptom onset and case

medRxiv preprint doi: https://doi.org/10.1101/2020.01.28.20019299.this version posted February 17, 2020. The copyright holder for this preprint (which was not certified by peer review) is the author/funder, who has granted medRxiv a license to display the preprint in perpetuity.
It is made available under a CC-BY-NC-ND 4.0 International license .

detection (*12*,*13*). Given the uncertainty in these estimates, we also provide estimates assuming shorter and longer delays in the lag between infection and case reporting (Appendix Table 3).

We ran 10 chains in parallel. Trace plot and diagnosis confirmed the convergence of MCMC chains with posterior median and 95% CrI estimates as follows:

- Epidemic growth rate, λ: 0.095 (0.072 - 0.111), corresponding to an epidemic doubling time of incident cases of 7.31 (95% CrI 6.26 - 9.66) days;
- Number of initial cases in Wuhan on December 1, 2019: 7.78 (95% CrI 5.09 - 18.27);
- Basic reproductive number, R_0: 1.90 (95% CrI 1.47 - 2.59);
- Cumulative number of infections in Wuhan by January 22, 2020: 12,400 (95% CrI 3,112–58,465);
- Case detection rate by January 22, 2020: 8.95% (95% CrI 2.22% - 28.72%). This represents the ratio between the 425 confirmed cases in Wuhan during this period (*22*) and our estimate that 4,747 (95% CrI 1,480–19,151) cumulative infections occurred by January 12, 2020 (i.e., ≥10 days before the quarantine to account for the typical lag between infection and case detection).

medRxiv preprint doi: https://doi.org/10.1101/2020.01.28.20019299.this version posted February 17, 2020. The copyright holder for this preprint (which was not certified by peer review) is the author/funder, who has granted medRxiv a license to display the preprint in perpetuity.
It is made available under a CC-BY-NC-ND 4.0 International license .

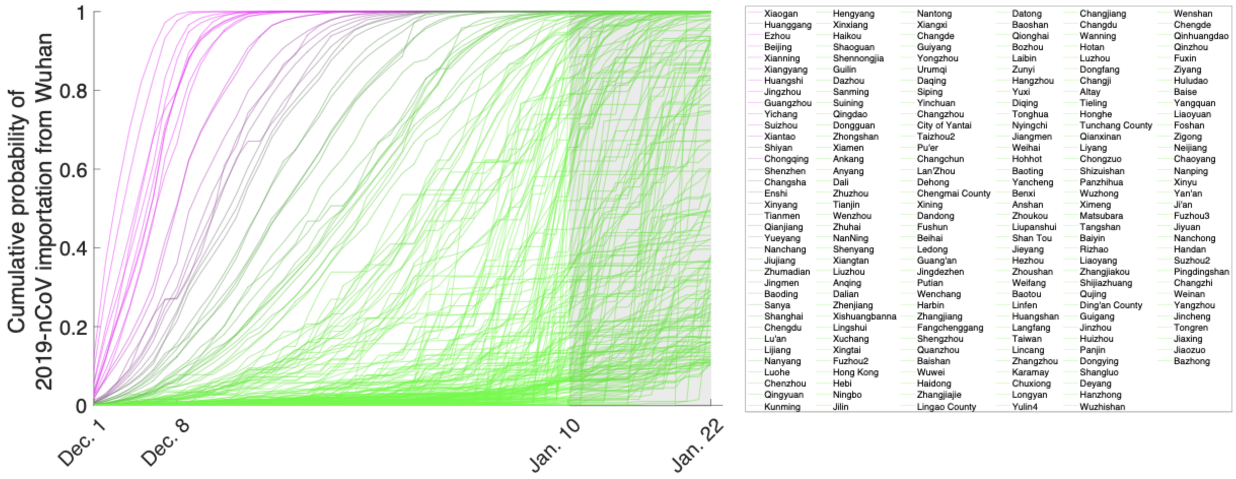

Figure S1. The risk for introduction of 2019 novel coronavirus (COVID-19) from Wuhan to other cities in China before the January 23, 2020 quarantine of Wuhan. Lines indicate probabilities that at ≥1 person infected with COVID-19 in Wuhan arrived in a listed city by the date indicated on the x-axis. The estimates were calculated by using mobility data collected from the location-based services of Tencent (https://heat.qq.com) during December 10, 2017–January 24, 2018, the timeframe that corresponds to the Spring Festival travel period of December 8, 2019–January 22, 2020. All cities with an expected importation probability >10% by January 22, 2020 (n = 212) are shown.

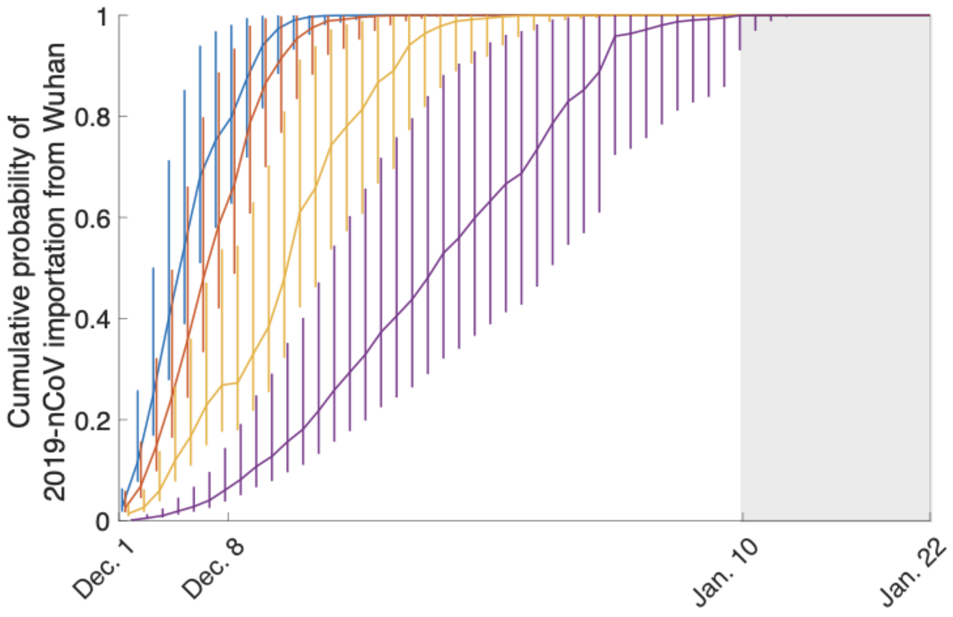

Figure S2. Uncertainty analysis representing the number of 2019 novel coronavirus (COVID-19) exposures in Wuhan per day. Lines show the probability that ≥1 transportation of COVID-19 infection occurred from Wuhan to Beijing, Guangzhou, Shenzhen, and Shanghai during December 8, 2020–January 22, 2020. Error bars indicate 95% credible intervals.

medRxiv preprint doi: https://doi.org/10.1101/2020.01.28.20019299.this version posted February 17, 2020. The copyright holder for this preprint (which was not certified by peer review) is the author/funder, who has granted medRxiv a license to display the preprint in perpetuity.
It is made available under a CC-BY-NC-ND 4.0 International license .

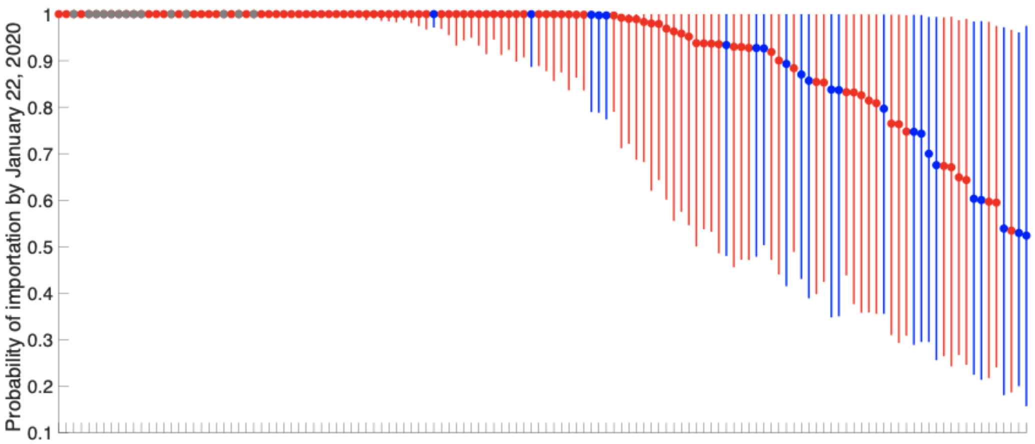

Figure S3. Risk for transportation of 2019 novel coronavirus (COVID-19) from Wuhan to 130 cities in China by January 23, 2020. All cities represented have mean importation probability >50%. As of January 26, 2020, 82.3% (107/130) of these cities had reported cases. Grey circles indicate cities that were included in the quarantine as of January 24, 2020. Red circles indicate cities outside the quarantine area with confirmed cases; blue circles indicate cities outside the quarantine area without confirmed cases as of January 26th, 2020.

Table S1. Cases of 2019 novel coronavirus detected outside of China*

Country/State	City	Date, 2020
Thailand	Bangkok	Jan 8
Thailand	Bangkok	Jan 17
Thailand	Bangkok	Jan 19
Thailand	Bangkok	Jan 21
Thailand	Chiang Mai	Jan 21
Nepal	Kathmandu	Jan 9
Vietnam	Hanoi	Jan 13
United States	Chicago	Jan 13
United States	Seattle	Jan 15
Singapore		Jan 21
Korea	Seoul	Jan 19

medRxiv preprint doi: https://doi.org/10.1101/2020.01.28.20019299.this version posted February 17, 2020. The copyright holder for this preprint (which was not certified by peer review) is the author/funder, who has granted medRxiv a license to display the preprint in perpetuity.
It is made available under a CC-BY-NC-ND 4.0 International license .

Korea	Seoul	Jan 22
Japan	Tokyo	Jan 18
Japan	Tokyo	Jan 19
Taiwan	Taipei	Jan 20
Taiwan	Taipei	Jan 21
Taiwan	Taipei	Jan 21
Australia	Sydney	Jan 18
Australia	Sydney	Jan 20

*As of January 22, 2020.

medRxiv preprint doi: https://doi.org/10.1101/2020.01.28.20019299.this version posted February 17, 2020. The copyright holder for this preprint (which was not certified by peer review) is the author/funder, who has granted medRxiv a license to display the preprint in perpetuity.
It is made available under a CC-BY-NC-ND 4.0 International license .

Table S2. Sensitivity analysis for the delay between infection and case confirmation, assuming that cases were confirmed either 6 d, 10 d (baseline), or 14 d after infection

Delay (*D*) from infection to case reporting	Posterior median (95% CrI)
D = 6 d	
Epidemic doubling time, d	6.79 (5.88 - 8.64)
Initial number of cases on December 1, 2019, i_0	7.95 (5.10 - 18.43)
Basic reproduction number, R_0	1.98 (1.54 - 2.71)
Cumulative cases by January 22, 2020	17,376 (4,410 - 80,915)
Cumulative cases by January 16, 2020 (*D* = 6 d before January 22, 2020)	9,362 (2,696 - 39,705)
Reporting rate through January 22, 2020	4.54% (1.07% - 15.8%)
D = 10 d	
Epidemic doubling time, d	7.31 (6.26 - 9.66)
Initial number of cases on December 1, 2019, i_0	7.78 (5.09 - 18.27)
Basic reproduction number, R_0	1.90 (1.47 - 2.59)
Cumulative cases by January 22, 2020	12,400 (3,112–58,465)
Cumulative cases by January 16, 2020 (*D* = 6 d before January 22, 2020)	4,747 (1,480–19,151)
Reporting rate through January 22, 2020	8.95% (2.22% - 28.72%)
D = 14 d	
Epidemic doubling time, d	7.64 (6.49 - 10.36)
Initial number of cases on December 1, 2019, i_0	7.62 (5.09 - 18.13)
Basic reproduction number, R_0	1.86 (1.44 - 2.52)
Cumulative cases by January 22, 2020	10,229 (2,564 - 48,681)
Cumulative cases by January 16, 2020 (*D* = 6 d before January 22, 2020)	2,805 (957 - 10,758)
Reporting rate through January 22, 2020	15.15% (3.95% - 44.41%)

Table S3. Mobility between Wuhan and 369 cities in China during December 3, 2016–January 24, 2017*

ID	City	Total trips	From Wuhan	To Wuhan	2016 population, millions
1	Xiaogan	9,646,286	5,333,682	4,312,604	4.90
2	Huanggang	7,786,732	4,436,928	3,349,804	6.32
3	Xianning	3,987,334	2,149,524	1,837,810	2.53
4	Ezhou	3,921,153	1,956,195	1,964,958	1.07

medRxiv preprint doi: https://doi.org/10.1101/2020.01.28.20019299.this version posted February 17, 2020. The copyright holder for this preprint
(which was not certified by peer review) is the author/funder, who has granted medRxiv a license to display the preprint in perpetuity.
It is made available under a CC-BY-NC-ND 4.0 International license .

5	Beijing	3,858,883	1,508,938	2,349,945	21.73
6	Jingzhou	3,439,123	2,216,479	1,222,644	5.70
7	Xiangyang	3,160,473	1,959,413	1,201,060	5.64
8	Huangshi	2,787,922	1,521,685	1,266,237	2.47
9	Guangzhou	2,555,286	705,205	1,850,081	14.04
10	Yichang	2,266,974	1,420,349	846,625	4.13
11	Shenzhen	1,675,478	188,316	1,487,162	11.91
12	Suizhou	1,536,742	934,564	602,178	2.20
13	Xiantao	1,492,596	856,578	636,018	1.15
14	Shiyan	1,252,190	897,666	354,524	3.41
15	Chongqing	1,177,096	720,442	456,654	30.48
16	Enshi	869,910	610,937	258,973	4.56
17	Tianmen	716,794	447,408	269,386	1.29
18	Changsha	644,273	318,784	325,489	7.65
19	Shanghai	571,458	72,150	499,308	24.20
20	Xinyang	564,841	338,180	226,661	6.44
21	Qianjiang	489,747	288,200	201,547	0.96
22	Jingmen	408,465	269,703	138,762	2.90
23	Yueyang	352,512	185,672	166,840	5.68
24	Zhumadian	316,181	214,425	101,756	6.99
25	Nanchang	301,903	123,239	178,664	5.37
26	Jiujiang	229,539	106,873	122,666	4.85
27	Baoding	205,124	126,334	78,790	11.63
28	Nanyang	173,653	127,666	45,987	10.07
29	Hengyang	155,591	32,443	123,148	7.29
30	Luohe	153,337	103,153	50,184	2.64
31	Sanya	151,726	29,147	122,579	0.75
32	Lijiang	121,669	33,825	87,844	1.29
33	Dazhou	120,983	120,983	0	5.60
34	Luan	117,242	53,698	63,544	4.77
35	Qingyuan	116,218	35,704	80,514	3.85
36	Chengdu	113,938	50,532	63,406	15.92
37	Kunming	108,452	46,613	61,839	6.73
38	Chenzhou	102,565	18,274	84,291	4.71
39	Guilin	100,723	92,078	8,645	5.01
40	Shaoguan	94,847	11,483	83,364	2.96
41	Shijiazhuang	93,102	70,128	22,974	10.78
42	Ankang	81,065	81,065	0	2.66
43	Xinxiang	73,246	54,707	18,539	5.74

medRxiv preprint doi: https://doi.org/10.1101/2020.01.28.20019299.this version posted February 17, 2020. The copyright holder for this preprint (which was not certified by peer review) is the author/funder, who has granted medRxiv a license to display the preprint in perpetuity.
It is made available under a CC-BY-NC-ND 4.0 International license .

44	Shennongjia	66,818	37,240	29,578	0.08
45	Suining	64,847	43,223	21,624	3.30
46	Haikou	64,774	30,848	33,926	2.24
47	Shenyang	64,258	33,663	30,595	8.29
48	Hanzhong	58,082	58,074	8	3.45
49	Anyang	57,825	38,146	19,679	5.13
50	Dongguan	57,672	44,125	13,547	8.26
51	Liuzhou	56,640	43,180	13,460	3.96
52	Zhuzhou	53,890	27,321	26,569	4.02
53	Handan	52,175	42,872	9,303	9.49
54	Fuzhou2	50,264	11,069	39,195	7.57
55	Sanming	48,697	36,007	12,690	2.55
56	NanNing	47,505	33,242	14,263	7.06
57	Xingtai	44,627	33,727	10,900	7.32
58	Xuchang	44,397	41,839	2,558	4.38
59	Anqing	41,590	17,398	24,192	4.61
60	Dali	40,710	17,524	23,186	3.56
61	Yongzhou	40,530	40,530	0	5.47
62	Xiamen	40,039	14,993	25,046	3.92
63	Qingdao	36,803	21,919	14,884	9.20
64	Nanchong	33,778	33,764	14	6.40
65	Pingdingshan	30,833	25,945	4,888	4.98
66	Tieling	30,807	13,535	17,272	2.65
67	Putian	30,488	21,972	8,516	2.89
68	Zhuhai	30,263	20,698	9,565	1.68
69	Wenzhou	29,609	15,634	13,975	9.18
70	Jiaozuo	26,455	26,445	10	3.55
71	Guangan	25,597	24,288	1,309	3.26
72	Nantong	22,577	7,753	14,824	7.30
73	Xiangtan	22,283	7,879	14,404	2.84
74	Langfang	21,900	7,301	14,599	4.62
75	Tianjin	21,343	12,018	9,325	15.62
76	Zhenjiang	21,092	17,499	3,593	3.18
77	Suzhou2	20,366	0	20,366	10.65
78	Huludao	19,114	18,044	1,070	2.55
79	Jincheng	18,326	18,318	8	2.32
80	Siping	17,782	3,610	14,172	3.20
81	Dalian	17,190	6,147	11,043	6.99
82	Zhongshan	17,181	14,989	2,192	3.23

medRxiv preprint doi: https://doi.org/10.1101/2020.01.28.20019299.this version posted February 17, 2020. The copyright holder for this preprint (which was not certified by peer review) is the author/funder, who has granted medRxiv a license to display the preprint in perpetuity.
It is made available under a CC-BY-NC-ND 4.0 International license .

83	Shangluo	17,033	16,740	293	2.37
84	Beihai	16,142	6,120	10,022	1.64
85	Changzhi	14,729	14,729	0	3.44
86	Bazhong	14,705	14,705	0	3.31
87	Hebi	14,173	9,224	4,949	1.61
88	Xishuangbanna	11,767	6,146	5,621	1.17
89	Hong Kong	11,453	5,823	5,630	7.45
90	Zhoukou	11,066	11,066	0	8.82
91	Urumqi	10,893	10,058	835	3.52
92	Harbin	10,110	5,991	4,119	10.98
93	Ningbo	9,964	5,272	4,692	7.88
94	Weinan	9,743	9,743	0	5.37
95	Changchun	9,379	6,040	3,339	7.51
96	Laibin	9,200	8,652	548	2.20
97	Panjin	9,130	8,398	732	1.44
98	Xiangxi	8,616	2,506	6,110	2.64
99	City of Yantai	8,223	4,390	3,833	7.06
100	Yuxi	7,895	5,513	2,382	2.38
101	Tangshan	7,604	7,152	452	7.84
102	Lingshui	7,477	1,792	5,685	0.36
103	Xining	7,414	5,460	1,954	2.33
104	Liyang	7,291	7,291	0	3.63
105	Hezhou	7,274	7,274	0	2.04
106	Hangzhou	7,112	797	6,315	9.19
107	Nanping	7,053	3,854	3,199	2.66
108	Yinchuan	6,789	3,364	3,425	2.08
109	Changzhou	6,761	6,761	0	4.71
110	Zigong	6,705	6,681	24	2.78
111	Fushun	6,576	5,816	760	2.07
112	Puer	6,335	3,781	2,554	2.62
113	Taizhou2	6,269	2,362	3,907	6.08
114	Changde	6,131	4,946	1,185	5.84
115	Jinzhou	6,034	5,919	115	3.06
116	Chengde	5,937	5,786	151	3.53
117	Yangzhou	5,840	5,840	0	4.49
118	Qujing	5,396	5,041	355	6.08
119	Yangquan	5,313	5,269	44	1.40
120	Anshan	5,308	4,044	1,264	3.61
121	Guiyang	5,183	3,207	1,976	4.70

medRxiv preprint doi: https://doi.org/10.1101/2020.01.28.20019299.this version posted February 17, 2020. The copyright holder for this preprint (which was not certified by peer review) is the author/funder, who has granted medRxiv a license to display the preprint in perpetuity.
It is made available under a CC-BY-NC-ND 4.0 International license .

122	Zhangjiajie	5,157	4,112	1,045	1.53
123	Quanzhou	5,127	1,705	3,422	8.58
124	Jian	5,126	0	5,126	4.92
125	Wuwei	4,965	4,679	286	1.82
126	Ledong	4,807	3,014	1,793	0.53
127	Liaoyang	4,554	4,255	299	1.84
128	Jiangmen	4,550	4,439	111	4.54
129	LanZhou	4,154	2,226	1,928	3.71
130	Qinhuangdao	4,147	3,883	264	3.09
131	Ziyang	3,971	3,933	38	2.54
132	Jingdezhen	3,971	1,916	2,055	1.65
133	Diqing	3,933	1,123	2,810	0.41
134	Shengzhou	3,871	1,134	2,737	0.96
135	Dehong	3,645	1,735	1,910	1.29
136	Panzhihua	3,536	2,197	1,339	1.24
137	Neijiang	3,526	3,493	33	3.75
138	Foshan	3,422	3,157	265	7.46
139	Zhangjiang	3,377	1,426	1,951	7.27
140	Qionghai	3,287	1,321	1,966	0.51
141	Hohhot	3,278	2,905	373	3.09
142	Luzhou	3,155	2,974	181	4.31
143	Dandong	3,136	2,165	971	2.41
144	Deyang	3,135	2,962	173	3.52
145	Baoshan	3,114	1,767	1,347	2.61
146	Fangchenggang	2,967	1,486	1,481	0.93
147	Chuxiong	2,966	2,419	547	2.74
148	Datong	2,881	1,914	967	3.42
149	Zunyi	2,775	1,544	1,231	6.23
150	Jilin	2,464	1,031	1,433	4.24
151	Haidong	2,421	1,062	1,359	1.45
152	Baotou	2,378	1,947	431	2.86
153	Chengmai County	2,301	905	1,396	0.59
154	Huangshan	2,226	959	1,267	1.38
155	Benxi	2,166	1,886	280	1.71
156	Wenchang	2,087	1,124	963	0.56
157	Liupanshui	2,086	589	1,497	2.91
158	Lingao County	2,085	1,349	736	0.52
159	Daqing	2,062	715	1,347	2.76
160	Bozhou	2,031	1,014	1,017	0.48

medRxiv preprint doi: https://doi.org/10.1101/2020.01.28.20019299.this version posted February 17, 2020. The copyright holder for this preprint (which was not certified by peer review) is the author/funder, who has granted medRxiv a license to display the preprint in perpetuity.
It is made available under a CC-BY-NC-ND 4.0 International license .

161	Honghe	1,960	1,262	698	4.68
162	Lincang	1,901	927	974	2.52
163	Yancheng	1,855	790	1,065	7.24
164	Shan Tou	1,847	786	1,061	5.58
165	Fuzhou3	1,846	0	1,846	4.00
166	Zhangjiakou	1,845	1,743	102	4.43
167	Yiyang	1,820	1,365	455	4.43
168	Dongying	1,794	1,624	170	2.13
169	Tonghua	1,792	749	1,043	2.17
170	Jieyang	1,765	940	825	6.09
171	Dongfang	1,759	894	865	0.44
172	Huizhou	1,745	1,694	51	4.78
173	Weihai	1,744	677	1,067	2.82
174	Wanning	1,741	792	949	0.57
175	Jiyuan	1,555	1,461	94	0.73
176	Longyan	1,535	508	1,027	2.63
177	Changjiang	1,535	953	582	0.23
178	Zhoushan	1,474	796	678	1.16
179	Xinyu	1,471	0	1,471	1.17
180	Nyingchi	1,448	260	1,188	0.20
181	Weifang	1,372	930	442	9.36
182	Qianxinan	1,371	514	857	2.84
183	Baishan	1,347	674	673	1.20
184	Changji	1,326	744	582	1.60
185	Chongzuo	1,203	777	426	2.07
186	Changdu	1,181	369	812	0.68
187	Baoting	1,168	460	708	0.17
188	Hotan	1,146	671	475	2.14
189	Linfen	1,118	793	325	4.46
190	Tunchang County	1,090	489	601	0.27
191	Qitaihe	1,087	569	518	0.87
192	Fuxin	1,065	823	242	1.78
193	Zhangzhou	980	335	645	5.05
194	Yulin4	967	461	506	5.76
195	Shihezi	945	802	143	0.60
196	Matsubara	930	330	600	2.78
197	Jixi	923	553	370	1.84
198	Qinzhou	902	491	411	3.24
199	Haibei	900	577	323	0.28

medRxiv preprint doi: https://doi.org/10.1101/2020.01.28.20019299.this version posted February 17, 2020. The copyright holder for this preprint (which was not certified by peer review) is the author/funder, who has granted medRxiv a license to display the preprint in perpetuity.
It is made available under a CC-BY-NC-ND 4.0 International license .

200	Tongren	893	893	0	3.14
201	Dingan County	882	494	388	0.29
202	Altay	824	446	378	0.62
203	Wuzhishan	806	429	377	0.11
204	Ganzi	779	192	587	1.18
205	Karamay	760	392	368	0.42
206	Chaoyang	750	704	46	2.95
207	Baise	722	402	320	3.62
208	Nujiang	720	377	343	0.54
209	Aral	711	365	346	0.33
210	Tower	705	481	224	1.35
211	Wuzhong	705	429	276	1.39
212	Yingkou	704	348	356	2.44
213	Ningde	690	446	244	2.89
214	Shizuishan	672	481	191	0.80
215	Ordos	630	458	172	2.06
216	Ximeng	629	458	171	1.00
217	Shuangyashan	609	185	424	1.46
218	Leshan	585	313	272	3.27
219	Hainan	585	253	332	0.48
220	Baiyin	583	262	321	1.72
221	Chaozhou	570	230	340	2.65
222	Haixi	566	458	108	0.52
223	Chifeng	552	487	65	4.31
224	Yanbian	522	379	143	2.10
225	Yanan	520	492	28	2.25
226	Liaoyuan	512	352	160	1.18
227	Wenshan	500	282	218	3.62
228	Yili	496	419	77	4.62
229	Shannan	494	212	282	0.34
230	Rizhao	485	326	159	2.90
231	Maoming	480	172	308	6.12
232	Qiongzhong	479	287	192	0.23
233	Guigang	475	261	214	4.33
234	Shuozhou	455	249	206	1.77
235	Baisha	451	262	189	0.12
236	Xian	450	450	0	8.83
237	Meishan	446	219	227	3.00
238	Xingan League	439	91	348	1.60

medRxiv preprint doi: https://doi.org/10.1101/2020.01.28.20019299.this version posted February 17, 2020. The copyright holder for this preprint (which was not certified by peer review) is the author/funder, who has granted medRxiv a license to display the preprint in perpetuity.
It is made available under a CC-BY-NC-ND 4.0 International license .

239	Wulanchabu	434	332	102	2.11
240	Bayannaoer	423	275	148	1.68
241	Mianyang	398	288	110	4.81
242	Shigatse	397	288	109	0.72
243	Alxa League	389	286	103	0.25
244	Aksu	373	202	171	2.46
245	Wuhai	369	230	139	0.56
246	Tongliao	367	201	166	3.12
247	Wujiaqu	357	103	254	0.09
248	Bazhou	357	216	141	1.28
249	Qiannan	348	299	49	3.26
250	Yichun	332	29	303	1.10
251	Ali	326	178	148	0.10
252	Zhongwei	324	217	107	1.15
253	Jiaxing	321	45	276	4.61
254	Zhengzhou	319	83	236	9.72
255	Huangnan	318	142	176	0.27
256	Kashgar	309	177	132	4.21
257	White	306	253	53	1.91
258	Cangzhou	303	187	116	7.51
259	Qingyang	294	256	38	2.24
260	Bijie	265	227	38	6.64
261	Anshun	261	206	55	2.33
262	Zibo	241	134	107	4.69
263	Jiuquan	235	144	91	1.12
264	Nagqu	233	231	2	0.48
265	Dingxi	227	128	99	2.79
266	Hechi	220	107	113	3.50
267	Chizhou	214	191	23	1.44
268	Tumshuk	210	32	178	0.17
269	Yangjiang	204	96	108	2.53
270	Jinchang	203	147	56	0.47
271	Liangshan	199	84	115	4.82
272	Turpan	197	157	40	0.63
273	Hulunbeir	196	151	45	2.53
274	Jinzhong	187	18	169	3.35
275	Yaan	184	130	54	1.54
276	Pingliang	175	129	46	2.10
277	Golow	175	167	8	0.20

medRxiv preprint doi: https://doi.org/10.1101/2020.01.28.20019299.this version posted February 17, 2020. The copyright holder for this preprint (which was not certified by peer review) is the author/funder, who has granted medRxiv a license to display the preprint in perpetuity.
It is made available under a CC-BY-NC-ND 4.0 International license .

278	Daxinganling	158	45	113	0.44
279	Yulin2	155	72	83	3.38
280	Binzhou	146	69	77	3.89
281	Zhaoqing	143	112	31	4.08
282	Zhangye	143	52	91	1.22
283	Qiqihar	143	85	58	5.05
284	Linxia	142	58	84	2.03
285	Jiayuguan	130	55	75	0.25
286	Lishui	127	41	86	2.17
287	Suihua	121	81	40	5.21
288	Guyuan	119	99	20	1.22
289	Heyuan	110	37	73	3.08
290	Mudanjiang	110	59	51	2.63
291	Wuzhou	108	61	47	3.02
292	Kezhou	107	11	96	0.62
293	Luliang	107	11	96	3.85
294	Taiyuan	103	0	103	4.34
295	Tianshui	101	82	19	3.32
296	Heihe	99	38	61	1.64
297	Yushu	94	87	7	0.41
298	Baoji	94	94	0	3.78
299	Laiwu	94	65	29	1.38
300	Yunfu	93	44	49	2.48
301	Yingtan	88	9	79	1.16
302	Tongchuan	81	60	21	0.85
303	Pingxiang	76	0	76	1.91
304	Jiamusi	76	38	38	2.36
305	Shaoxing	76	44	32	4.99
306	Xinzhou	72	19	53	3.16
307	Shanwei	70	43	27	3.04
308	Dezhou	68	24	44	5.79
309	Jinhua	63	0	63	5.52
310	Meizhou	61	41	20	4.36
311	Hami	61	31	30	0.61
312	Lhasa	60	60	0	0.60
313	Yuncheng	59	42	17	5.31
314	Gannan	51	26	25	0.71
315	Liaocheng	36	0	36	6.04
316	Zhaotong	35	35	0	5.48

medRxiv preprint doi: https://doi.org/10.1101/2020.01.28.20019299.this version posted February 17, 2020. The copyright holder for this preprint (which was not certified by peer review) is the author/funder, who has granted medRxiv a license to display the preprint in perpetuity.
It is made available under a CC-BY-NC-ND 4.0 International license .

317	Jinan	30	30	0	7.23
318	Guangyuan	28	19	9	2.64
319	Hegang	26	19	7	1.04
320	Luoyang	21	0	21	6.80
321	Tongling	18	0	18	1.60
322	Chuzhou	17	0	17	4.04
323	Huzhou	16	0	16	2.98
324	Bozhou	13	7	6	5.10
325	Taian	11	0	11	5.64
326	Quzhou	10	0	10	2.16
327	Huaibei	10	0	10	2.21
328	Zaozhuang	9	0	9	3.92
329	Huaihua	8	0	8	4.92
330	Bengbu	7	0	7	3.33
331	Huainan	7	0	7	3.46
332	Xuancheng	6	0	6	2.60
333	Hengshui	6	0	6	4.45
334	Longnan	6	0	6	2.60
335	Hefei	0	0	0	7.87
336	Ganzhou	0	0	0	8.59
337	Shuanghe	0	0	0	0.05
338	Maanshan	0	0	0	2.78
339	Bazhou	0	0	0	0.94
340	Linyi	0	0	0	10.44
341	Beitun	0	0	0	0.08
342	Yibin	0	0	0	4.51
343	Shangqiu	0	0	0	7.28
344	Taizhou4	0	0	0	4.65
345	Shaoyang	0	0	0	7.32
346	Heze	0	0	0	8.62
347	Yichun	0	0	0	5.53
348	Wuxi	0	0	0	6.53
349	Fuyang	0	0	0	7.99
350	Yutian County, Xinjiang	0	0	0	0.22
351	Xuzhou	0	0	0	8.71
352	Suqian	0	0	0	4.88
353	Hetian County, Xinjiang	0	0	0	0.28
354	Huaian	0	0	0	4.89

medRxiv preprint doi: https://doi.org/10.1101/2020.01.28.20019299.this version posted February 17, 2020. The copyright holder for this preprint (which was not certified by peer review) is the author/funder, who has granted medRxiv a license to display the preprint in perpetuity.
It is made available under a CC-BY-NC-ND 4.0 International license .

355	Kaifeng	0	0	0	4.55
356	Nanjing	0	0	0	8.27
357	Loudi	0	0	0	3.89
358	Suzhou4	0	0	0	5.60
359	Macau	0	0	0	0.63
360	Jining	0	0	0	8.35
361	Qiandongnan	0	0	0	3.51
362	Kokodala	0	0	0	0.08
363	Xianyang	0	0	0	4.99
364	Lianyungang	0	0	0	4.50
365	Gejiu, Yunnan	0	0	0	0.47
366	Shangrao	0	0	0	6.75
367	Moyu County, Xinjiang	0	0	0	0.53
368	Wuhu	0	0	0	3.67
369	Sanmenxia	0	0	0	2.26

*Data derived from user geolocation data from Tencent (https://heat.qq.com). Cities are sorted according to the overall travel volume to and from Wuhan. These data also are available from github (https://github.com/ZhanweiDU/2019nCov.git).

medRxiv preprint doi: https://doi.org/10.1101/2020.01.28.20019299.this version posted February 17, 2020. The copyright holder for this preprint (which was not certified by peer review) is the author/funder, who has granted medRxiv a license to display the preprint in perpetuity.
It is made available under a CC-BY-NC-ND 4.0 International license .

References

1. Bogoch II, Watts A, Thomas-Bachli A, Huber C, Kraemer MUG, Khan K. Pneumonia of unknown etiology in Wuhan, China: potential for international spread via commercial air travel. J Travel Med. 2020 Jan 14 [Epub ahead of print]. PubMed https://doi.org/10.1093/jtm/taaa008

2. Wu JT, Leung K, Leung GM. Nowcasting and forecasting the potential domestic and international spread of the 2019-nCoV outbreak originating in Wuhan, China: a modelling study. Lancet (2020) https://doi.org/10.1016/S0140-6736(20)30260-9

3. Lai S, Bogoch II, Watts A, Khan K, Li Z, Tatem A. Preliminary risk analysis of 2019 novel coronavirus spread within and beyond China. https://www.worldpop.org/resources/docs/china/WorldPop-coronavirus-spread-risk-analysis-v1-25Jan.pdf

4. Li Q, Guan X, Wu P, Wang X, Zhou L, Tong Y, et al. Early transmission dynamics in Wuhan, China, of novel coronavirus–infected pneumonia. N Engl J Med. 2020 Jan 29 [Epub ahead of print] PubMed https://doi.org/10.1056/NEJMoa2001316

5. Huang C, Wang Y, Li X, Ren L, Zhao J, Hu Y, et al. Clinical features of patients infected with 2019 novel coronavirus in Wuhan, China. Lancet. 2020 Jan 24 [Epub ahead of print]. PubMed https://doi.org/10.1016/S0140-6736(20)30183-5

6. Imai N, Dorigatti I, Cori A, Donnelly C, Riley S, Ferguson NM. MRC Centre for Global Infectious Disease Analysis. News 2019-nCoV. Report 2: estimating the potential total number of novel coronavirus cases in Wuhan City, China. London: Imperial College London; 21 Jan 2020. [cited 2020 Jan 26]. https://www.imperial.ac.uk/media/imperial-college/medicine/sph/ide/gida-fellowships/2019-nCoV-outbreak-report-22-01-2020.pdf

7. Cauchemez S, Fraser C, Van Kerkhove MD, Donnelly CA, Riley S, Rambaut A, et al. Middle East respiratory syndrome coronavirus: quantification of the extent of the epidemic, surveillance biases, and transmissibility. Lancet Infect Dis. 2014;14:50–6. PubMed https://doi.org/10.1016/S1473-3099(13)70304-9

medRxiv preprint doi: https://doi.org/10.1101/2020.01.28.20019299.this version posted February 17, 2020. The copyright holder for this preprint (which was not certified by peer review) is the author/funder, who has granted medRxiv a license to display the preprint in perpetuity.
It is made available under a CC-BY-NC-ND 4.0 International license .

8. Donnelly CA, Ghani AC, Leung GM, Hedley AJ, Fraser C, Riley S, et al. Epidemiological determinants of spread of causal agent of severe acute respiratory syndrome in Hong Kong. Lancet. 2003;361:1761–6. PubMed https://doi.org/10.1016/S0140-6736(03)13410-1

9. Backer JA, Klinkenberg D, Wallinga J. The incubation period of 2019-nCoV infections among travellers from Wuhan, China. [medRxiv preprint of Infectious Diseases (except HIV/AIDS) January 28, 2020]. https://doi.org/10.1101/2020.01.27.20018986

10. Hay J. Turning nCoV case reports into infection incidence [cited 2020 Jan 31]. Github. https://github.com/jameshay218/case_to_infection

11. Lauer SA, Grantz KH, Bi Q, Jones FK, Zheng Q, Meredith H, et al. The incubation period of 2019-nCoV from publicly reported confirmed cases: estimation and application. medRxiv. February 04, 2020https://doi.org/10.1101/2020.02.02.20020016

12. World Health Organization. Disease outbreak news: novel coronavirus–Thailand (ex-China) [Internet] 14 Jan 2020 [cited 2020 Jan 27]. https://www.who.int/csr/don/14-january-2020-novel-coronavirus-thailand-ex-china/en

13. Ministry of Health, Labour and Welfare, China. Development of pneumonia associated with the new coronavirus [in Chinese]. [cited 27 Jan 2020]. https://www.mhlw.go.jp/stf/newpage_08906.html

14. Gutierrez B, Hill S, Kraemer M, Loskill A, Mekaru S, Pigott D, et al. Epidemiological and demographic data of confirmed cases in the 2019-nCoV outbreak. Github [cited 2020 Jan 31]. https://github.com/BoXu123/2019-nCoV-epiData

15. MOBS Lab. 2019 nCOV [Internet]. [cited 31 Jan 2020]. https://www.mobs-lab.org/2019ncov.html

16. Models of Infectious Disease Agent Study (MIDAS). Central resource of data and information in support of modeling research on the 2019 novel coronavirus (2019-nCoV). Google Docs [cited 2020 Feb 2]. https://docs.google.com/document/d/1pL6ogED0Qix08V0zjJbbNVhf6yf-xBShWVxLbXKVLXI/edit

17. Genomic epidemiology of novel coronavirus (nCoV). [cited 2020 Feb 2]. https://nextstrain.org/ncov

18. Wang L, Wu JT. Characterizing the dynamics underlying global spread of epidemics. Nat Commun. 2018;9:218. PubMed https://doi.org/10.1038/s41467-017-02344-z

medRxiv preprint doi: https://doi.org/10.1101/2020.01.28.20019299.this version posted February 17, 2020. The copyright holder for this preprint (which was not certified by peer review) is the author/funder, who has granted medRxiv a license to display the preprint in perpetuity.
It is made available under a CC-BY-NC-ND 4.0 International license .

19. Scalia Tomba G, Wallinga J. A simple explanation for the low impact of border control as a countermeasure to the spread of an infectious disease. Math Biosci. 2008;214:70–2. PubMed https://doi.org/10.1016/j.mbs.2008.02.009

20. Gautreau A, Barrat A, Barthélemy M. Global disease spread: statistics and estimation of arrival times. J Theor Biol. 2008;251:509–22. PubMed https://doi.org/10.1016/j.jtbi.2007.12.001

21. Chinese Center for Disease Control and Prevention. Epidemic update and risk assessment of 2019 novel coronavirus [cited 31 Jan 2020]. http://www.chinacdc.cn/yyrdgz/202001/P020200128523354919292.pdf

22. Real-time surveillance of pneumonia epidemics in China [Internet]. [cited 2020 Jan 27]. https://3g.dxy.cn/newh5/view/pneumonia?scene=2&clicktime=1579579384&enterid=1579579384&from=timeline&isappinstalled=0
